# Optimal Extent of Transhiatal Gastrectomy and Lymphadenectomy for the Stomach-Predominant Adenocarcinoma of Esophagogastric Junction: Retrospective Single-Institution Study in China

**DOI:** 10.3389/fonc.2018.00639

**Published:** 2019-01-21

**Authors:** Baoyu Zhao, Zhenzhan Zhang, Debin Mo, Yiming Lu, Yanfeng Hu, Jiang Yu, Hao Liu, Guoxin Li

**Affiliations:** ^1^Department of General Surgery, Nanfang Hospital, Southern Medical University, Guangzhou, China; ^2^Department of General Surgery, Shanxi Provincial People's Hospital, Taiyuan, China

**Keywords:** esophagogastric junction, Siewert II adenocarcinoma, Siewert's classification, Nishi's classification, transhiatal gastrectomy, lymphadenectomy

## Abstract

**Background:** The optimal extent of gastrectomy and lymphadenectomy for esophagogastric junction (EGJ) cancer is controversial. Our study aimed to compare the long-term survival of transhiatal proximal gastrectomy with extended periproximal lymphadenectomy (THPG with EPL) and transhiatal total gastrectomy with complete perigastric lymphadenectomy (THTG with CPL) for patients with the stomach-predominant EGJ cancer.

**Methods:** Between January 2004, and August 2015, 306 patients with Siewert II tumors were divided into the THTG group (*n* = 148) and the THPG group (*n* = 158). Their long-term survival was compared according to Nishi's classification. The Kaplan–Meier method and Cox proportional hazards models were used for survival analysis.

**Results:** There were no significant differences between the two groups in the distribution of age, gender, tumor size or Nishi's type (*P* > 0.05). However, a significant difference was observed in terms of pathological tumor stage (*P* < 0.05). The 5-year overall survival rates were 62.0% in the THPG group and 59.5% in the THTG group. The hazard ratio for death was 0.455 (95% CI, 0.337 to 0.613; log-rank *P* < 0.001). Type GE/E = G showed a worse prognosis compared with Type G (*P* < 0.05). Subgroup analysis stratified by Nishi's classification, Stage IA-IIB and IIIA, and tumor size ≤ 30 mm indicated significant survival advantages for the THPG group (*P* < 0.05). However, this analysis failed to show a survival benefit in Stage IIIB (*P* > 0.05).

**Conclusions:** Nishi's classification is an effective method to clarify the subdivision of Siewert II tumors with a diameter ≤ 40 mm above or below the EGJ. THPG with EPL is an optimal procedure for the patients with the stomach-predominant EGJ tumors ≤30 mm in diameter and in Stage IA-IIIA. For more advanced and larger EGJ tumors, further studies are required to confirm the necessity of THTG with CPL.

## Introduction

**Figure 1:**
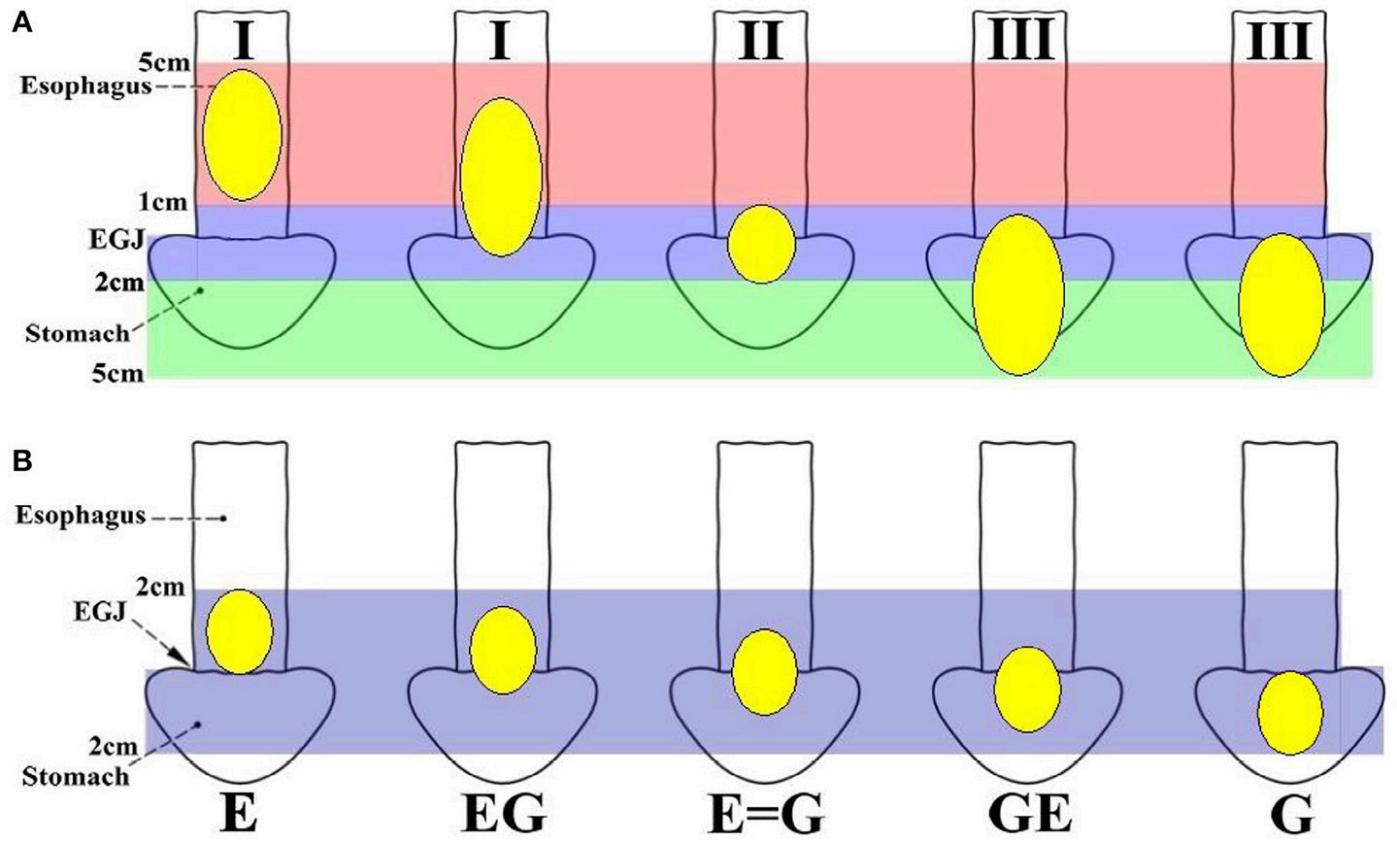
Definition and classification of EGJ cancer according to the epicenter location. **(A)** Siewert's classification defines three types of EGJ cancers with epicenters situated between 5 cm proximal and distal to the EGJ, regardless of tumor size. Type I tumors (lower oesophageal adenocarcinoma) are located 1–5 cm above the EGJ, irrespective of EGJ involvement. Type II tumors (cardia adenocarcinoma) are located 1 cm above to 2 cm below the EGJ. Type III tumors (subcardial gastric adenocarcinoma) are located 2–5 cm below the EGJ with involvement of the EGJ and distal esophagus. Siewert's classification Types I, II and III should also be described for adenocarcinoma located in the lower esophagus or at the EGJ. **(B)** Nishi's classification defines five types of EGJ cancer with 40 mm or less in dimension that have an epicenter within 2 cm proximal or distal to the EGJ, irrespective of histological type. The terms “E, EG, E = G, GE, and G” were used to describe subtype depending on the epicenter location at the oral “E” and anal “G” portions of the EGJ. EGJ, Esophagogastric Junction.

Epidemiological data show an increasing incidence of esophagogastric junction (EGJ) cancer (1–5). Because of the lack of a uniform definition and classification, EGJ cancer has sometimes been treated as distal esophageal cancer, sometimes as proximal gastric cancer, and sometimes as an entity separated from both esophageal and gastric cancer (6, 7). Obviously, EGJ cancer is distinguished from carcinomas of the lower esophagus or the upper stomach (6). Nevertheless, there are inconsistent prognoses among subtypes of EGJ cancer (8, 9). Siewert's classification (Figure 1A) (8, 9) defines three types of EGJ adenocarcinoma (Type I-III) with epicenters located within 5 cm proximal and distal to the anatomical cardia, regardless of tumor size. Type I tumors (lower-esophageal adenocarcinoma) are located 1–5 cm above the EGJ, irrespective of EGJ involvement. Type II tumors (cardia adenocarcinoma) are located between 1 cm above and 2 cm below the EGJ. Type III tumors (subcardial gastric adenocarcinoma) are located 2–5 cm below the EGJ with involvement of the EGJ and distal esophagus. In Japan, Nishi's classification (Figure 1B) (7, 8) was employed by the Japanese Classification of Esophageal Cancer and Gastric Cancer to define five types of EGJ cancer characterized by diameters of 40 mm or less and an epicenter within 2 cm proximal or distal from the EGJ, irrespective of histological type. The “E-G” terms of “E,” “EG,” “E = G,” “GE” and “G” were used to describe the subtype according to the epicenter location at the rostral and caudal portions of the EGJ. In fact, EGJ cancer based on Nishi's classification corresponds to Siewert Type II-True cardia cancer according to the Japanese Classification of Esophageal Cancer and Gastric Cancer (Figure 1) (7).

Surgical resection of the primary tumor plus adequate lymphadenectomy remains a mainstay of therapy for resectable EGJ tumors. Special attention should also be paid to the surgical procedure. Based on previous studies of Siewert's classification (6, 9), there is consensus on the surgical treatment for Type I (transthoracic esophagectomy) and Type III (transhiatal extended gastrectomy). However, there is no consensus over the extent of gastrectomy and lymphadenectomy that could be a standard of care for Type II based on Siewert's and Nishi's classifications (8–14).

Considering the discrepancies among the classifications and survival data, our study compared the long-term survival of transhiatal proximal gastrectomy with extended periproximal lymphadenectomy (THPG with EPL) and transhiatal total gastrectomy with complete perigastric lymphadenectomy (THTG with CPL). As EGJ cancer corresponds to the description of Siewert II tumors, Nishi's definition “E-G” can be used to classify the subdivision of Siewert II adenocarcinoma into tumors located above or below the EGJ.

## Patients and Methods

### Cohort

The study was approved by the Institutional Review Board of Nanfang Hospital, Southern Medical University. A total of 1918 patients with gastric or cardia adenocarcinoma underwent potentially curative gastrectomy at Nanfang Hospital, Southern Medical University, Guangzhou, China, between January 2004 and August 2015 according to the proposed standard for EGJ cancer from the Japanese gastric cancer classification (15) and treatment guideline (8, 16) (3rd and 4th Edition). The EGJ was defined as the border between the esophageal and gastric muscles. It was identified by one of the following clinical criteria: (a) the distal end of the longitudinal palisading small vessels in the lower esophagus at endoscopy, (b) the horizontal level of the angle of His shown by barium meal examination, (c) the proximal end of the longitudinal folds of the greater curve of the stomach shown at endoscopy or barium meal examination or (d) the level of the macroscopic caliber change of the resected esophagus and stomach. After the retrospective review of the institutional database including the medical records of these patients by two independent surgical oncologists, the following categories of patients were excluded from this study: 837 (43.6%) patients with distal gastric cancer and 733 (38.2%) patients with Siewert III, gastric upper and body cancer, transthoracic resection, squamous carcinoma, hospital deaths, surgical exploration only, chemotherapy alone, and endoscopic resection alone.

**Figure 2:**
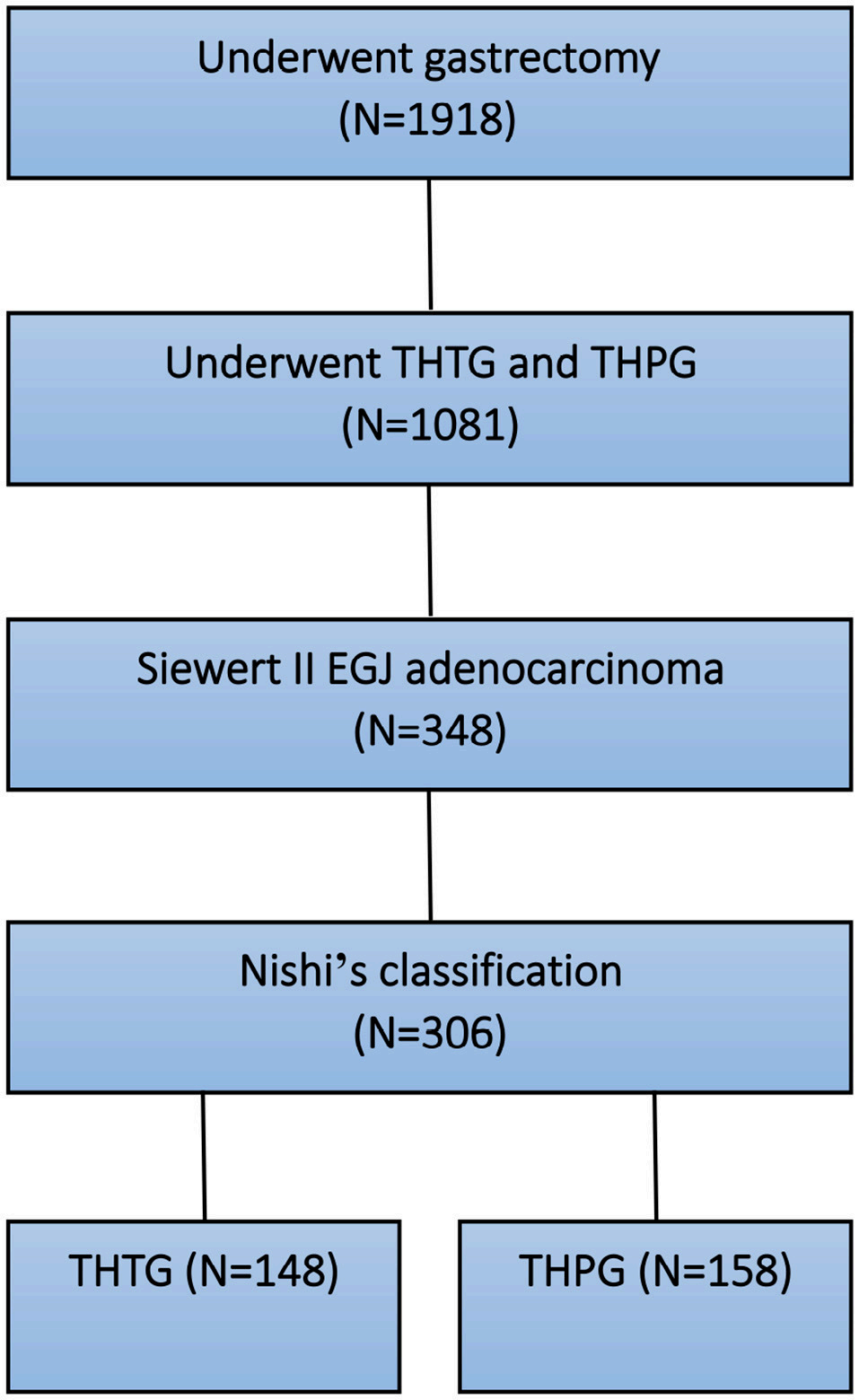
Selection and grouping diagram for the patients with EGJ adenocarcinoma. EGJ, Esophagogastric Junction; THTG, Transhiatal Total Gastrectomy; THPG, Transhiatal Proximal Gastrectomy.

After the above exclusions, 348 patients with Siewert II tumors were enrolled. As the EGJ cancer corresponded to Siewert II cancer according to Nishi's definition (7, 8), Nishi's classification “E-G” was used to clarify the subdivision of Siewert II adenocarcinoma into tumors located above or below the EGJ (Figure 1 and Table 1). Another 42 patients were excluded due to tumor size of >40 mm, R2 status or unavailability of follow-up data. Finally, 306 patients were eligible for this study. All tumors were classified as Type GE/E = G or Type G according to the epicenter location at the EGJ. The enrolled patients were divided into a THTG group and a THPG group based on the type of gastric resection with lymph node dissection (Figure 2). Histological type was defined as adenocarcinoma according to Siewert's classification (17, 18). All the patients provided written informed consent. All relevant data, including demographic information, location, “E-G” subtype, lymphadenectomy and gastrectomy were collected according to the tentative standard for junctional cancer of the Japanese Gastric Cancer (8) and Esophageal Cancer Society (7) and were in accordance with the ethics review board at the Southern Medical University and ethical standards of the Declaration of Helsinki.

**Table 1:** EGJ cancer corresponds to Siewert II adenocarcinoma according to the epicenter location.

**Definition**	**Epicenter**	**Oral E(Esophagus “+”)**					**EGJ**	**Anal G(Stomach “-”)**				
	**Zone**	**+5 cm**	**+2 cm**		**+1 cm**		**0 cm**	**−1 cm**		**−2 cm**		**−5 cm**

Siewert (AC)	+5 ~ −5 cm	1~5 cm			1~2 cm							2~5 cm
Type I	E	Y	Y		N		N					
Type I	EG	Y	Y		Y		Y	N				
Type II	GE		N		Y		Y	Y		Y		N
Type III	G		N		Y		Y	Y		Y		Y
Nishi (AC/SC)	+2 ~ −2 cm	Size ≤ 4 cm	2 cm		1 cm		EGJ	1 cm		2 cm		
Type E	E	N	Y		Y		Y	N				
Type EG	EG		N	Y	Y		Y	Y	N			
Type E = G	E = G		N		Y		Y	Y		N		
Type GE	GE				N	Y	Y	Y		Y	N	
Type G	G				N		Y	Y		Y		N

*A quantitative comparison of epicenter location between Siewert's and Nishi's classifications. EGJ cancer corresponds to Siewert II adenocarcinoma according to the epicenter location. The calibrated and colored cells only illustrated the exact location of the epicenter in the EGJ region. In cancers located at the EGJ, the oral and anal portions of the EGJ are described as “E” and “G,” respectively. The terms “E, EG, E=G, GE, and G” can be used depending on the epicenter location. EGJ, esophagogastric junction; AC, adenocarcinoma; SC, squamous carcinoma; Y, involvement; N, no involvement*.

### Transhiatal Gastrectomy and Lymphadenectomy

The surgical procedures THPG with EPL and THTG with CPL were routinely undertaken according to the local surgeon's evaluation and preference. All the resection procedures of the parahiatal and lower mediastinal nodes included only the lymph nodes around the distal esophagus, which was accessed transhiatally. Since the two surgical procedures included different extents of lymphadenectomy and gastrectomy, the patients underwent precise assessment of tumor stages, with abdominal, and thoracic CT scans, endoscopy, upper gastrointestinal contrast, and laboratory tests before surgery. Patients who had positive lavage cytology and macroscopic peritoneal metastasis were considered incurable.

The THPG with EPL procedure consisted of proximal gastrectomy and extended proximal perigastric nodal dissection along the upper and middle portions of the stomach, esophageal hiatus, distal esophagus and suprapancreatic area, while the THTG with CPL procedure consisted of total gastrectomy and complete perigastric nodal dissection along the total perigastric portion, esophageal hiatus, distal esophagus, and suprapancreatic area.

### Follow-Up

Long-term survival was the primary endpoint in this study. As of August 2015, the median follow-up duration was 69.2 months (95% CI 42.5–59.5). Thirty patients in the THTG group and 8 in the THPG group were lost to follow-up during this study. In this study, the overall survival was measured from the date of surgery to the date of death from any cause or to lost follow-up. All in-hospital deaths and deaths within 1 month of surgery were excluded from the analysis. Patients who were still alive at the end of the study, lost to follow-up, or died of any cause were marked as censored data. Tumor staging was adapted from the 8th edition of AJCC/UICC system (AJCC, American Joint Committee on Cancer /UICC, Union for International Cancer Control).

### Statistical Analysis

Data are presented as the means ± standard deviations for continuous variables and as number (%) for categorical variables. Continuous and categorical variables were compared using *t*-tests and chi-square tests, respectively. Survival curves were estimated using the Kaplan-Meier method and compared with the log rank test. Cox proportional hazards regression was used to identify the predictors associated with overall survival. Statistical significance was defined as a two-sided *P-*value < 0.05. These statistical analyses were performed using IBM SPSS, version 25.0 (IBM, Armonk, NY, USA).

## Results

### Comparison of Siewert's and Nishi's Classification

A comparison of Siewert's and Nishi's classifications is presented in Table 1. EGJ cancer corresponded to Siewert II tumor with a diameter of ≤40 mm. Nishi's classification is an effective method to clarify the subdivision of Siewert II tumors with a diameter of ≤40 mm into tumors located above or below the EGJ. The stomach- and esophagus-predominant cancers were designated as having their epicenters located at the rostral and caudal portions of the EGJ, respectively (Figure 1 and Table 1).

### Demographics and Pathologic Characteristics

A total of 306 patients with Siewert II adenocarcinoma of 40 mm or less in diameter were included in the retrospective single-institution study. The demographics and pathological characteristics of the two groups are provided in Table 2. Both THTG and THPG groups showed comparable demographics, including age, gender, body mass index, tumor size, pathological N0, E-G type, and extent of lymphadenectomy (all *P* > 0.05). Statistically significant differences were found in terms of pathological depth, positive nodal status, and TNM category (*P* < 0.05). The distribution of Eastern Cooperative Oncology Group performance scores (ECOG-PS) differed significantly between THTG and THPG (*P* < 0.05). THTG tumors were significantly more advanced in terms of Bormann and differentiation type (*P* < 0.05).

**Table 2:** Demographics and pathologic characteristics.

**Characteristics**	**Total**	**THTG with CPL**	**THPG with EPL**	***P***
	***N* = 306 (%)**	***n* = 148 (%)**	***n* = 158 (%)**
Age(mean±SD)	57 ± 10.85	58 ± 10.10	56 ± 10.42	0.140
< 65 years	222 (72.5%)	106 (34.6%)	116 (37.9%)	0.798
≥65 years	84 (27.5%)	42 (13.7%)	42 (13.7%)
Gender				0.662
Male	248 (81.0%)	118 (38.6%)	130 (42.5%)	
Female	58 (19.0%)	30 (9.8%)	28 (9.2%)
Body mass index	21.63 ± 3.13	21.54 ± 3.23	21.81 ± 2.95	0.591
Nishi's Classification				0.733
Type GE/E = G	148 (48.4%)	70 (22.9%)	78 (25.5%)	
Type G	158 (51.6%)	78 (25.5%)	80 (26.1%)
ECOG-PS				0.000
PS 0	229 (74.8%)	92 (30.2%)	144 (47.1%)	
PS 1-2	64 (20.9%)	50 (16.3%)	14 (4.6%)
Tumor size (mm)	25.44 ± 18.03	26.60 ± 18.12	24.61 ± 18.00	0.450
≤ 30mm	218 (71.4%)	90 (29.6%)	128 (41.8%)	0.874
>30mm	88 (28.6%)	38 (12.2%)	50 (16.3%)
Bormann type				0.015
Type 1-2	106 (34.6%)	40 (13.1%)	66 (21.6%)
Type 3-4	142 (46.4%)	80 (26.1%)	62 (20.3%)
Type 5	58 (19.0%)	28 (9.2%)	30 (9.8%)
Differentiation				0.000
G1-G2	132 (43.1%)	34 (11.1%)	98 (32.0%)
G3-G4	166 (54.2%)	110 (35.9%)	56 (18.3%)
Gx	8 (2.6%)	4 (1.3%)	4 (1.3%)
pT category				0.000
pT1(M/SM)	8 (2.6%)	4 (1.3%)	4 (1.3%)
pT2(MP)	22 (7.2%)	4 (1.3%)	18 (5.9%)
pT3(SS)	10 (3.3%)	4 (1.3%)	6 (2.0%)
pT4a(SE)	230 (75.2%)	100 (32.7%)	130 (42.5%)
pT4b(SI)	36 (11.8%)	36 (11.8%)	0 (0%)
pN category				0.000
pN0(0)	68 (22.2%)	28 (9.2%)	40 (13.1%)
pN(+)	238 (77.8%)	120 (39.2%)	118 (38.6%)
pN1(1-2)	62 (20.3%)	24 (7.8%)	38 (12.4%)
pN2(3-6)	86 (28.1%)	34 (11.1%)	52 (17.0%)
pN3a(7-15)	58 (19.0%)	34 (11.1%)	24 (7.8%)
pN3b(≥16)	32 (10.5%)	28 (9.2%)	4 (1.3%)
pTNM category				0.000
Stage-I	18 (5.9%)	6 (2.0%)	12 (3.3%)
IA	6 (2.0%)	4 (1.3%)	2 (0.7%)
IB	12 (3.9%)	2 (0.7%)	10 (3.3%)
Stage-II	60 (19.6%)	22 (7.2%)	38 (5.6%)
IIA	8 (2.6%)	2 (0.7%)	6 (2.0%)
IIB	52 (17.0%)	20 (6.5%)	32 (10.5%)
Stage-III	228 (74.5%)	120 (39.2%)	108 (39.7%)
IIIA	130 (42.5%)	48 (15.7%)	82 (26.8%)
IIIB	54 (17.6%)	32 (10.5%)	22 (7.2%)
IIIC	44 (14.4%)	40 (13.1%)	4 (1.3%)
Lymphadenectomy				0.183
D2/D2+	250 (81.7%)	116 (37.9%)	134 (43.8%)
D1/D1+	56 (18.3%)	32 (10.5%)	24 (7.8%)
Chemotherapy	176 (57.5%)	96 (31.4%)	80 (26.1%)	0.430
Neoadjuvant	40 (13.0%)	24 (7.8%)	16 (5.2%)
Adjuvant	136 (44.4%)	72 (23.5%)	64 (20.9%)

*Tumor stage according to the American Joint Committee on Cancer, 8th Edition. ECOG-PS, Eastern Cooperative Oncology Performance Score; G1-G2, Well-differentiation; G3-G4, Poor-differentiation; Gx, Unknown; THTG, Transhiatal Total Gastrectomy; THPG, Transhiatal Proximal Gastrectomy; EPL, Extended Periproximal Lymphadenectomy; CPL, Complete Perigastric Lymphadenectomy*.

### Long-Term Overall Survival

**Figure 3:**
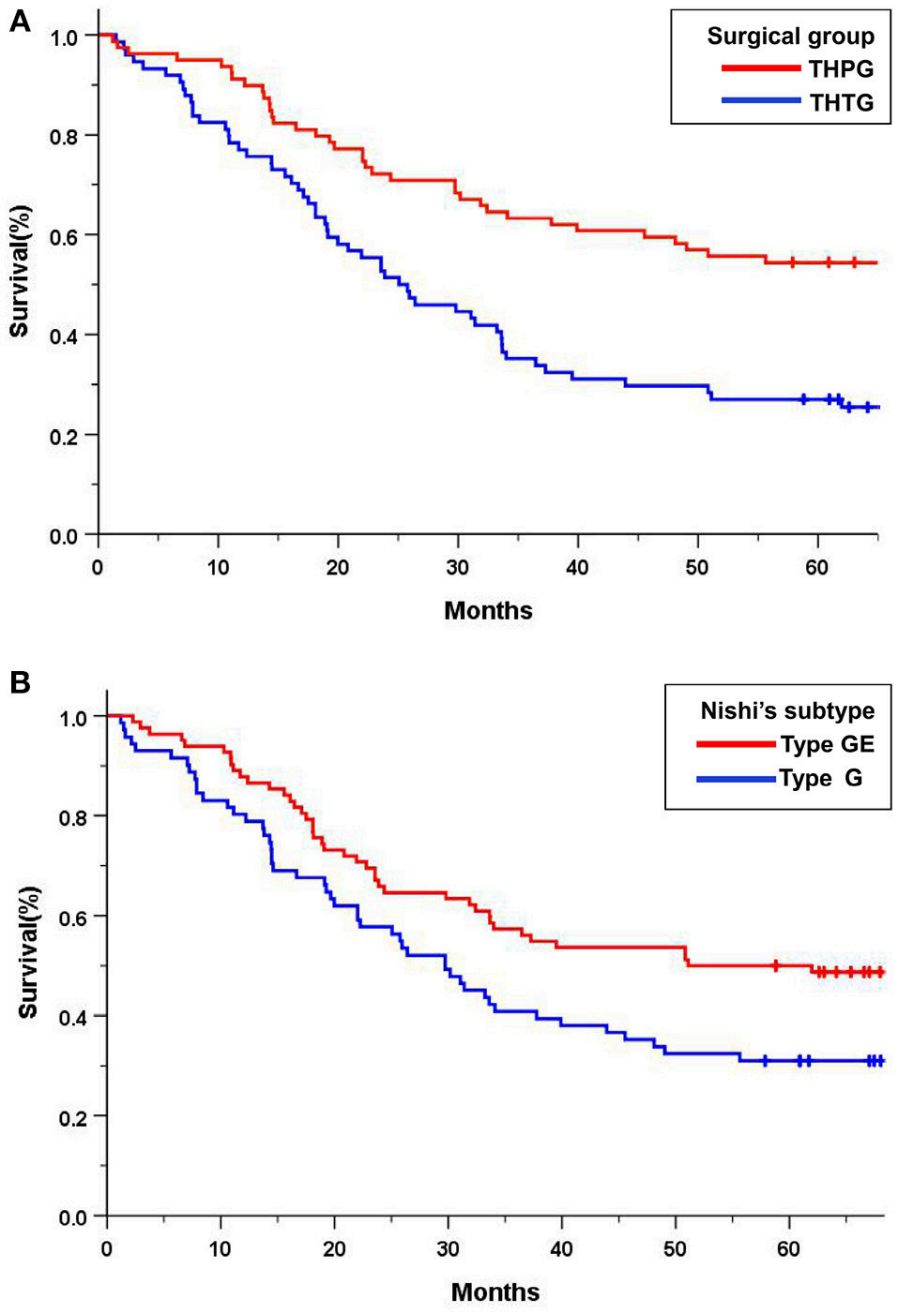
Kaplan-Meier curves of overall survival in all the patients by treatment group and Nishi's classification. **(A)** THTG vs. THPG (HR = 0.455, 95%CI 0.337–0.613, log-rank *P* = 0.000); **(B)**. Type GE vs. Type G (HR = 0.604, 95%CI 0.450–0.811, log-rank *P* = 0.001). HR, Hazard Ratio; CI, Confidence Ratio; EGJ, Esophagogastric Junction; THTG, Transhiatal Total Gastrectomy; THPG, Transhiatal Proximal Gastrectomy; Type GE, and Type G, “E-G” subtype according to Nishi's classification.

The median overall survival was 50.9 months (95% CI 42.5–59.5) for patients assigned to the THTG group and 81.1 months (95% CI 72.7–89.5) for those assigned to the THPG group. Thirty patients were lost to follow-up in the THTG group and 8 in the THPG group. The 5-year overall survival was 62.0% for all the patients in the THPG group and 59.5% in the THTG group (*P* = 0.000). The hazard ratio of death for THPG compared with THTG was 0.455 (95% CI, 0.337 to 0.613; log-rank *P* < 0.001; Figure 3A). Type GE tumors had a worse survival, the hazard ratio for death was 0.604 (95% CI, 0.450-0.811; log-rank *P* = 0.001; Figure 3B) compared with Type G tumors.

**Figure 4:**
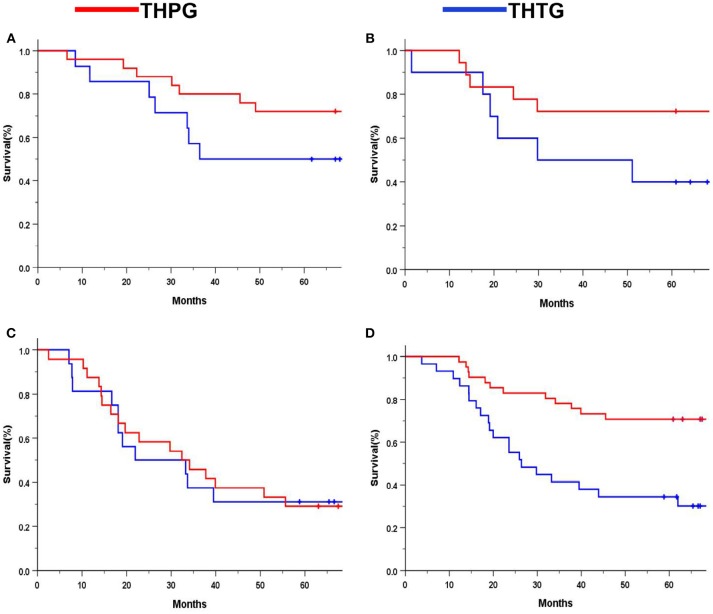
Kaplan-Meier curves of subgroup analysis by treatment. **(A)** Stage IA-IIB (log-rank *P* = 0.044); **(B)** Stage IIIA (log-rank *P* = 0.029); **(C)** Stage IIIB (log-rank *P* = 0.211); **(D)** Tumor ≤ 30 mm (log-rank *P* = 0.000). EGJ, Esophagogastric Junction; THTG, Transhiatal total gastrectomy; THPG, Transhiatal proximal gastrectomy.

**Figure 5:**
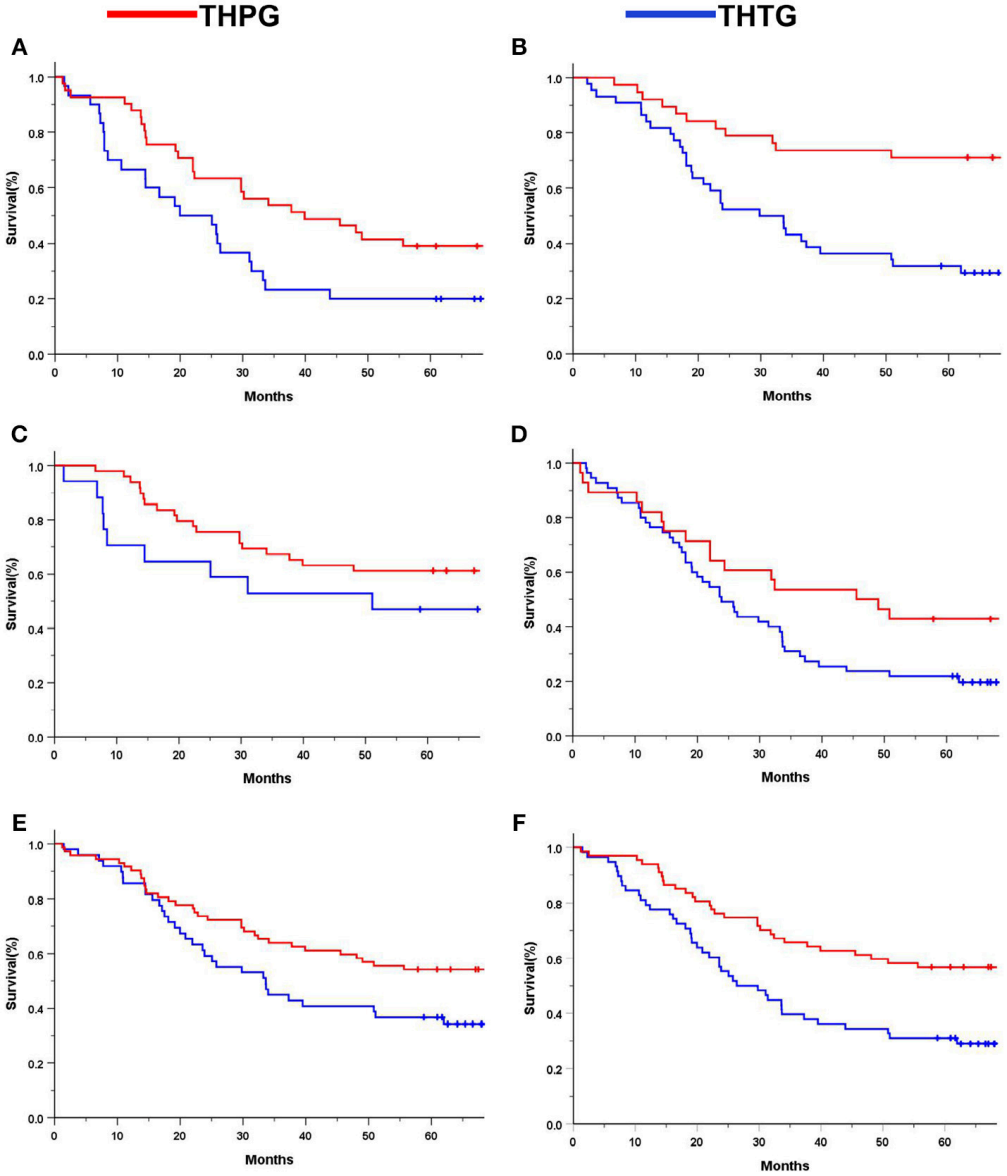
Kaplan-Meier curves of subgroup analysis by treatment. **(A)**. Type GE (log-rank *P* = 0.002); **(B)** Type G (log-rank *P* = 0.000); **(C)** Well-differentiation (log-rank *P* = 0.068); **(D)** Poor-differentiation (log-rank *P* = 0.005); **(E)** ECOG-PS0 (log-rank *P* = 0.003); **(F)** D2/D2+ (log-rank *P* = 0.000). EGJ, Esophagogastric Junction; THTG, Transhiatal Total Gastrectomy; THPG, Transhiatal Proximal Gastrectomy; Type GE and Type G, “E-G” Subtype according to Nishi's classification; ECOG-PS, Eastern Cooperative Oncology Performance Score; D2/D2+, D2/D2+ lymphadenectomy according to the EGJ cancer with Type GE.

Subgroup analysis indicated significant survival advantages based on the subgroups of Stage IA-IIB (*P* = 0.044; Figure 4A) and IIIA (*P* = 0.029; Figure 4B), and tumors ≤ 30 mm (*P* = 0.000; Figure 4D) in favor of the THPG group compared with the THTG group but failed to show an advantage for Stage IIIB (*P* = 0.211; Figure 4C). In addition, more detailed subgroup analysis stratified by Type GE (*P* = 0.002) and Type G (*P* = 0.000), Well-differentiation (*P* = 0.068) and Poor-differentiation (*P* = 0.005), ECOG-PS0 (*P* = 0.003), and D2/D2+ lymphadenectomy (*P* = 0.000) also yielded similar findings in favor of the THPG group (Figures 5A–F).

The potential predictors associated with OS in univariate analyses were analyzed by multivariate analysis using a proportional hazards model, to identify independent predictors associated with OS (Table 3). Ultimately, the following factors were determined to be the negative predictors: Type GE, THTG, D1/D1+, tumor size > 30 mm, Bormann type 3-4, pTNM category greater than Stage IIA. The strongest surgical predictors that were associated with OS were THPG and D2/D2+ lymphadenectomy.

**Table 3:** Cox proportional hazards models.

**Predictors**	**Category**	**Univariate**		**Multivariable**	
		**HR (95%CI)**	***P***	**HR (95%CI)**	***P***
Age(years)	<65	1		1
	≥65	1.443 (1.056–1.972)	0.021	1.567 (0.929–2.644)	0.092
Performance score	PS 0	1		1
	PS 1–2	2.328 (1.683–3.220)	<0.001	0.534 (0.273–1.045)	0.067
Nishi's definition	Type GE	1		1
	Type G	0.604 (0.450–0.811)	0.001	0.508 (0.316–0.816)	0.005
Lymphadenectomy	D2/D2+	1		1
	D1/D1+	1.893 (1.340–2.675)	<0.001	2.328 (1.357–3.993)	0.002
Gastrectomy	TH–TG	1		1
	TH–PG	0.455 (0.337–0.613)	<0.001	0.468 (0.290–0.755)	0.002
Tumor size	≤ 30 mm	1		1
	>30 mm	2.222 (1.504–3.284)	<0.001	2.028 (1.326–3.100)	0.001
Bormann type	Type 1–2	1		1
	Type 3–4	1.809 (1.276–2.565)	0.001	2.186 (1.272–3.755)	0.005
	Type 5	1.971 (1.302–2.983)	0.001	4.216 (1.645–10.810)	0.003
Differentiation	G1–G2	1		1
	G3–G4	2.184 (1.588–3.003)	<0.001	1.628 (0.961–2.759)	0.070
pTNM–category	I–II	1	<0.001	1
	IIIA	2.062 (1.336–3.182)	0.001	1.946 (1.012–3.741)	0.046
	IIIB	2.995 (1.846–4.860)	<0.001	2.065 (1.032–4.132)	0.040
	IIIC	5.067 (3.096–8.294)	<0.001	2.115 (0.951–4.701)	0.066
pT–category	pT1–T2	1		—	—
	pT3	2.609 (0.905–7.524)	0.076	
	pT4a	2.920 (1.431–5.960)	0.003	
	pT4b	5.861 (2.681–12.813)	<0.001	
pN–category	pN0	1		—	—
	pN1	0.934 (0.516–1.691)	0.823	
	pN2	3.099 (1.935–4.962)	<0.001	
	pN3a	4.133 (2.526–6.760)	<0.001	
	pN3b	4.634 (2.652–8.098)	<0.001	
	pN1–3	2.634 (1.713–4.051)	<0.001	

*Tumor stage according to the American Joint Committee on Cancer, 8th Edition. ECOG-PS, Eastern Cooperative Oncology Performance Score; G1-G2, Well-differentiation; G3-G4, Poor-differentiation; HR, Hazard Ratio; CI, Confidence Interval; THTG, Transhiatal Total Gastrectomy; THPG, Transhiatal Proximal Gastrectomy*.

## Discussion

The optimal extent of gastrectomy with lymphadenectomy in the EGJ cancer has been controversial. In this retrospective single institution study, we compared the long-term survival of transhiatal proximal gastrectomy with extended periproximal lymphadenectomy (THPG with EPL) and total gastrectomy with complete perigastric lymphadenectomy (THTG with CPL) for patients with the stomach-predominant EGJ cancer according to Nishi's classification. The findings demonstrated that THPG with EPL showed an advantage in survival compared with THTG with CPL for patients with EGJ tumors ≤30 mm in diameter and in Stage IA-IIIA. However, for more advanced and larger EGJ cancers (Stage IIIB), no survival benefit was demonstrated. As Type GE/E = G had a worse prognosis compared with Type G, Nishi's classification was an effective method to clarify the subdivision of Siewert II tumors with a diameter of ≤40 mm into tumors located above or below the EGJ line. We concluded that THPG with EPL should be considered as a specific modality to optimize the extent of gastrectomy and lymphadenectomy for individual patients with EGJ cancer ≤30 mm in dimension and in Stage IA-IIIA. However, for more advanced and larger EGJ tumors, further studies are required to confirm the necessity of THTG with CPL.

For EGJ tumors, tumor location, histological type, and tumor size are important for the selection of the surgical procedure in clinical practice. Therefore, an effective classification is particularly important. In most studies (6–8, 11) of EGJ cancer, the Siewert's classification is commonly used because it facilitates the selection of the surgical approach, especially for Type I and III tumors. However, there are considerable difficulties in the surgical approach, and the extent of gastrectomy and lymphatic dissection for Type II tumors (10), regardless of tumor size. This may be, in part, due to the imprecise definition of the gastric cardia, and also because it is difficult to identify its subtype when the tumor body is larger than 50 mm (7). Therefore, Nishi's definition was used, which determined that the diameter of the EGJ tumor was 40 mm or less and EGJ area was 2 cm above and below the cardia regardless of histological type. Considering the epicenter location at the rostral and caudal portion of the EGJ, EGJ cancer corresponds to Siewert II cancer according to the Japanese Classification of Esophageal Cancer and Gastric Cancer (Figure 1, Table 1). Based on the comparison between Siewert's and Nishi's classifications, we properly used the Nishi's classification to clarify the subdivision of Siewert II tumors into tumors located above or below the EGJ. Because 71.4% of Siewert II tumors had a diameter of ≤30 mm in this study, the finding showed a marked survival difference between Type GE/E = G and G. Nishi's classification is effective in clarifying the subclass of Siewert II tumors with a diameter of ≤40 mm into tumors located above or below the EGJ (Figure 1, Table 1).

Regarding differences between Siewert II and III tumors, comparisons of cases in Western and Eastern countries had inconsistent findings (1, 19, 20). In a prospective study from Germany by Siewert and colleagues, an almost equal distribution of Siewert I, II, and III EGJ cases was observed (21). In contrast, in Eastern countries, such as Japan and China, Type II-III tumors are more common (5, 16). In the current study, Type GE/E = G and G were almost equally common (48.4 vs. 51.6%, respectively).

Multiple studies (21–24) have shown varied prognosis among patients with Type II and III cancers, and these observations were likely due to incomparable baseline characteristics (25). Some reports (13, 23) demonstrated that Siewert II cancer had a better survival than Siewert III cancer. In contrast, the other findings (20) showed no survival difference, and even a worse survival (26). However, in the present study, the univariate analyses confirmed that Type GE/E = G had a marked association with a worse prognosis compared with Type G. This finding was subsequently confirmed by the multivariate analysis. This finding actually suggested that esophageal invasion was a risk predictor for overall survival. It is important to highlight the limitation that the patients with tumors of >40 mm in diameter were excluded in the study according to Nishi's classification (27, 28), because these tumors undergoing THPG or even THTG are extremely likely to be incurable by surgery alone (29, 30).

The surgical approach for Siewert II and III tumors usually depends on the Siewert's classification (10). For patients with Siewert II disease, the RCT from the Netherlands (9) demonstrated that the transthoracic compared to the transhiatal approach was not associated with a survival benefit. The JCOG9502 RCT trial (11) also confirmed that the transthoracic approach should be abandoned due to increased morbidity and mortality, but no survival advantage was observed above the transhiatal approach for Siewert II and III cancers with esophageal invasion of ≤30 mm. Therefore, the extent of gastrectomy and lymphadenectomy seemed to be a controversial issue for Siewert II (6, 7, 12, 14, 18, 26–29).

In Asia, THTG with a more extensive lymphadenectomy is a fairly common procedure for EGJ tumors, regardless of tumor depth and size (31). However, unlike THTG, the THPG procedure removed only the periproximal nodes except for the lower perigastric lymph nodes. Therefore, we can conclude that the THTG based on a more extensive lymphadenectomy should provide a survival advantage over THPG. However, the current findings indicted no survival advantage in favor of THTG with CPL. The 5-year overall survival was 62.0% for the THPG group and 59.5% for the THTG group. As this was a retrospective single-institution study with strict inclusion criteria, some characteristics was not well balanced between the groups. After adjustment of nine baseline variables (age, performance score, Nishi's type, tumor size, Borrmann type, differentiation, gastrectomy, lymphadenectomy, and pathological TN stage) with the use of Cox regression analysis, the finding was essentially unchanged. A more detailed subgroup analysis further confirmed the survival advantage of THPG for patients with EGJ tumors ≤30 mm in dimension and in Stage IA-IIIA (*P* < 0.05), which consisted of N0-2 categories with six positive nodes or less. This result may suggest that the THPG with EPL procedure not only removes the nodes likely to be violated in the Siewert II cancer with Stage IA-IIIA but also may reduce short- and long-term complications. The individualized subclass may not require THTG with CPL from the viewpoint of sufficient lymph node dissection (8, 12, 14, 28, 32–34).

Interestingly, no survival advantage to support either THPG or THTG in the subgroup of Stage IIIB consisted of pN3a-3b, while the IIIC subgroup consists of pN3b. N3a and N3b exceeding six positive nodes were found to be the most powerful risk predictor in the univariate analysis of this study. In light of the results, we can hypothesize that N3 represents an extensive nodal metastasis, not just an increase in the numbers of positive lymph nodes because positive nodes were harvested from each involved station. Therefore, the potential benefit of THTG depends not only on the number of positive nodes but also on the propensity for extensive nodal metastasis (29, 32, 35). This finding is consistent with recent studies (12, 14, 32, 36) showing that more than 90% of all the metastatic nodes were distributed in the periproximal portion of the stomach, esophageal hiatus, distal esophagus, and suprapancreatic area. In contrast, the incidences of metastasis around the lower perigastric portion and greater curvature lymph nodes were <1%, even in patients with high dissection rates. Additionally, Siewert II/III cancer involving parapyloric nodes has a poor prognosis, similar to stage IV disease. This may explain why the THTG with CPL showed no survival advantage over THPG with EPL in the present study (28, 35). We can conclude that THTG with CPL along the lower perigastric nodes seems unlikely to offer significant survival benefits compared with THPG with EPL for the patients with Siewert II adenocarcinoma with a diameter of ≤40 mm because of rare nodal metastasis. The EPL along the proximal portion of the stomach, esophageal hiatus, distal esophagus, and suprapancreatic area may be the most essential step for the EGJ cancer, because D2/D2+ lymphadenectomy based on Type E-G of Nishi's definition was one of the strongest surgical predictors associated with OS for this study.

In this respective study, our results clearly showed that THTG with CPL provided no survival advantage over THPG with EPL for Siewert II adenocarcinomas with a diameter of ≤40 mm, at least, for EGJ tumors ≤30 mm in diameter and in Stage IA-IIIB. In addition, both Japanese and Dutch RCTs clearly confirmed that transthoracic lymphadenectomy provided no survival benefits over transhiatal lymphadenectomy for Siewert II adenocarcinoma. We did not aim to demonstrate that THTG with CPL was worse, since THTG with CPL may be a more thorough lymphadenectomy for more advanced and larger EGJ cancers accompanied by the distal perigastric lymph node metastasis (28). Accordingly, THPG with EPL should be considered as a specific modality to optimize the extent of gastrectomy and lymph node dissection for the individualized subgroup of EGJ cancer.

There are some limitations to this study. As it was a retrospective single-institution study with strict inclusion criteria, some characteristics were not well balanced between the groups. A selection bias concerning the surgical procedures might exist because limited surgical materials would be difficult to obtain. Despite this, the findings were sufficient to conclude that THPG with EPL was an optimal procedure for patients with the stomach-predominant EGJ tumors ≤30 mm in diameter and in Stage IA-IIIA.

## Conclusions

Nishi's classification is effective to clarify the subdivision of Siewert II tumors with a diameter of 40 mm or less into tumors located above or below the EGJ. Transhiatal proximal gastrectomy is an optimal procedure for patients with EGJ tumors ≤30 mm in diameter and in Stage IA-IIIA. However, for more advanced and larger EGJ tumors, further studies are required to confirm the necessity of transhiatal total gastrectomy.

## Ethics Approval and Consent to Participate

This study was carried out in accordance with the recommendations of the proposed standard for junctional cancer from the Japanese Gastric Cancer and Esophageal Cancer Society. This study was approved by the ethics review board of Nanfang Hospital, Southern Medical University. All study subjects provided written informed consent in accordance with the Declaration of Helsinki.

## Availability of Supporting Data

The datasets generated and/or analyzed during the current study are not publicly available due to other ongoing studies but are available from the corresponding authors on reasonable request.

## Author Contributions

BZ, GL, and HL made substantial contributions to the conception and design, acquisition of data, or analysis and interpretation of data. BZ, ZZ, DM, YL, JY, and YH contributed in drafting the manuscript or critically revising it for important intellectual content. All the authors approved the final manuscript submitted. Each author participated sufficiently in the work to take public responsibility for appropriate portions of the content and agreed to be accountable for all aspects of the work in ensuring that questions related to the accuracy or integrity of any part of the work are appropriately investigated and resolved. BZ should be considered as the first author. GL and HL contribute equally and should be considered as co-corresponding author.

### Conflict of Interest Statement

The authors declare that the research was conducted in the absence of any commercial or financial relationships that could be construed as a potential conflict of interest.

## Abbreviations

EGJ: esophagogastric junction
THTG: transhiatal total gastrectomy
THPG: transhiatal proximal gastrectomy
EPL: extended periproximal lymphadenectomy
CPL: complete perigastric lymphadenectomy
HR: hazard ratio
CI: confidence interval
OS: overall survival
RCT: randomized clinical trial.
